# Reproductive factors and *NOS3* variant interactions in primary open-angle glaucoma

**Published:** 2011-09-30

**Authors:** Jae Hee Kang, Janey L. Wiggs, Jonathan Haines, Wael Abdrabou, Louis R. Pasquale

**Affiliations:** 1 Channing Laboratory, Department of Medicine, Harvard Medical School and Brigham & Women’s Hospital, Boston, MA; 2 Department of Ophthalmology, Harvard Medical School, Massachusetts Eye and Ear Infirmary, Boston, MA; 3 Center for Human Genetics Research, Vanderbilt University School of Medicine, Nashville, TN

## Abstract

**Purpose:**

To investigate whether associations with the nitric oxide synthase gene (*NOS3*) variants and risk of primary open-angle glaucoma (POAG) depend on female reproductive factors.

**Methods:**

Two functional and two tagging single nucleotide polymorphisms (SNPs; T-786C: rs2070744, Glu298Asp: rs1799983, rs7830, and rs3918188) were evaluated in a nested case-control study from the Nurses’ Health Study (women followed 1980 – 2002). Participants were aged ≥40 years and Caucasian, who were followed biennially with update information on reproductive factors. We included 374 Nurses’ Health Study (NHS) cases and 1,085 controls, matched on age and eye exam at the matched cases’ diagnosis dates. Relative risks (RRs) were estimated using multivariable conditional logistic regression.

**Results:**

Among women with age at menarche <13 years, compared with the CC homozygotes of the rs3918188 tagging SNP, the wild-type AA homozygotes were at significantly reduced risk of POAG (RR=0.31, 95% CI=0.16, 0.59); however, for women with age at menarche ≥13 years, the SNP was not associated with POAG (p-interaction=0.0007). Among parous women with 3+ children, carriers of the minor variant (T) allele of the functional *Glu298Asp* SNP were at increased risk, while among parous women with 1–2 children, they were not (p-interaction=0.003). No significant interactions between *NOS3* SNPs and oral contraceptive use in POAG were detected.

**Conclusions:**

These data provide further support for the notion that *NOS3* genotype - female reproductive health interactions are important in POAG pathogenesis.

## Introduction

Nitric oxide synthase 3 (NOS3) is an enzyme that catalyzes the production of nitric oxide (NO), which modulates the tone of luminal structures with adjacent smooth muscle tissue [1]. NOS3 may play a role in the etiology of glaucoma; it is found in the human outflow pathway [2] and the vasculature supplying retinal ganglion cells [3], which may affect the regulation of intraocular pressure (IOP) and blood flow to the optic nerve, respectively.

Dysfunction in the ocular vascular endothelium and in the trabecular meshwork cells could lead to primary open angle glaucoma (POAG). Feke and Pasquale [4] documented unstable retinal blood flow after physiologic alterations in ocular perfusion pressure, which implicates dysfunction in NO-mediated responses [5-7]. NO also influences trabecular meshwork cell volume and outflow facility [8-10] that in turn influences IOP, an established POAG risk factor.

Reproductive hormones such as estrogen may play a role in modulating IOP and the risk of POAG in women [11-19]. One mechanism for this protective effect might be that estrogen upregulates *NOS3*, thereby enhancing the release of nitric oxide [20-23] to regulate blood flow to the optic nerve and the outflow facility. Previously, we observed that early menopause was associated with an increased risk of POAG and current use of estrogen with progestin was associated with a reduced risk of high tension POAG [24]. Also, we observed associations with nitric oxide synthase gene (*NOS3*) variants that were significantly stronger in women than in men and evidence of stronger associations with *NOS3* variants among current users of postmenopausal hormones (PMH) compared to non-current users in relation to high-tension POAG [25].

In this study, we evaluated whether the associations with *NOS3* variants and the risk of POAG depended on the status of reproductive factors of age at menarche, parity, oral contraceptive use. We used data from a large case-control study nested within the Nurses’ Health Study (NHS) where for cases, the exposure information was collected before any diagnoses of POAG.

## Methods

### Study population

The NHS was initiated in 1976, when 121,700 female US registered nurses (aged 30–55 years) returned a questionnaire on health-related information [26]. Participants have been followed with biennial questionnaires to update information on lifestyle factors and newly diagnosed illnesses, such as glaucoma [27]. Follow-up rates were high (>95% of the total possible person-time through 2002). The Human Research Committees of Brigham & Women’s Hospital and Massachusetts Eye and Ear Infirmary approved this study.

### Blood and cheek sample collection

In 1989–1990, blood samples were collected from 32,826 (27%) women. In 2001–2004, among 29,684 women who did not provide a blood sample, buccal cell samples were collected.

Blood samples were collected using sodium heparin as the anticoagulant. Samples were mailed back within 26 h of blood draw, immediately centrifuged, aliquoted into buffy coat components, red blood cells, and plasma, and stored in liquid nitrogen freezers for later retrieval. Buccal cell samples were collected using a single “swish-and-spit” procedure (women were provided a small bottle of mouthwash and a small cup with a cap seal and were asked to swish the mouthwash and then spit into the cup, which was returned by mail). Within a week of receipt, samples were processed and DNA was extracted.

### Case and control ascertainment

We first identified POAG cases with the biennial questionnaires, where we asked whether participants received an eye exam with a diagnosis of “glaucoma.” For participants self-reporting glaucoma, we sought permission to retrieve the medical records. We contacted the diagnosing eye care provider for all prior visual field (VF) tests and for the completion of a glaucoma questionnaire; this questionnaire included items for maximal IOP, the status of the filtration apparatus, optic nerve structural information, prior ophthalmic surgery, and any VF loss. Relevant medical records were accepted in lieu of questionnaires. All confirmatory information from questionnaires/medical records and VFs were evaluated in a standardized manner by a glaucoma specialist (L.R.P.). We previously demonstrated our case definition is valid by showing appropriate positive associations with a family history of glaucoma and with African heritage [28].

We included as cases only participants determined to have “definite” or “probable” POAG. For definite POAG cases (70% of case), slit lamp biomicroscopy showed no sign of pigment dispersion syndrome, uveitis, exfoliation syndrome, trauma, or rubeosis in either eye; gonioscopy demonstrated that the angles were open in both eye; and ≥2 reliable VFs showing reproducible defects consistent with glaucoma had to be available. For probable POAG cases, the same slit lamp exam and VF criteria were also required, but documentation of pupil dilation without subsequent adverse events was accepted if gonioscopy was not performed. For VFs, there was no requirement for the type of perimetry performed; however, in 95% of cases, full static threshold testing was completed, and in <1% of cases, kinetic VFs were used. For static threshold or suprathreshold tests, we regarded the VF reliable if the fixation loss rate was ≤33%, the false positive rate was ≤20% and the false negative rate was ≤20%. For kinetic VFs, we considered the field reliable unless the examiner noted otherwise.

We included 374 cases and 1,085 control subjects who were at least 40 years in age and Caucasian. The controls were matched on sample type (blood or cheek cell), year of birth, ethnicity (Latino or not), and they were required to have had an eye exam in the same period as when the matched case was diagnosed. Approximately three controls per case were matched to each case, using incidence density sampling.

### Genotyping

We genotyped two functional single nucleotide polymorphisms (SNPs) (T −786C: rs2070744 and Glu298Asp: rs1799983) and two tagging SNPs (rs3918188, and rs7830). The tagging SNPs corresponded to the *NOS3* linkage disequilibrium (LD) blocks, and were selected using Haploview (version 4.1) according to the HapMap data (release 22) from the Utah residents with Northern and Western European ancestry (CEU) population [29], with the minimum minor allele frequency set to 0.01. The two functional SNPs and the two tagging SNPs (rs3918188 and rs7830) captured the majority (88%) of alleles at r^2^ greater than 0.8 across the whole gene, including the 5′- and 3′-untranslated regions.

For DNA extraction, 50 μl of buffy coat or 20 μl of cheek cells were diluted with 150 μl of PBS and processed via the QIAmp (QIAGEN Inc., Chatsworth, CA), 96 spin blood kit protocol. A quantitative PCR approach (TaqMan assay; Applied Biosystems, Inc.; Foster City, CA) was used for genotyping, according to the manufacturer’s instructions. The RT–PCR amplification of whole genome amplified DNA was performed in 96 well plates with a sequence detection system (ABI Prism© 7000 Sequence Detection System; Applied Biosystems Inc., Foster City, CA). The thermal cycler (model 2720; Applied Biosystems Inc., Foster City, CA) was set at the following parameters: 50 °C for 2 min, 95 °C for 10 min, 92 °C for 15 s, and 58 °C for 1 min for a total of 60 cycles. The genotyping success rate was over 90%. Plates that passed quality control measures (including Hardy–Weinberg equilibrium tests) were included, and in 5% of samples that underwent repeat genotyping, there was >95% concordance on genotyping calls.

### Assessment of reproductive factors

#### Age at menarche and age at menopause

From 1976, participants were asked about the age at menarche and when menstrual periods ceased permanently. Additional questions were asked of menopausal women, including type of menopause (natural, surgical, or radiation) and, if surgical menopause, the number of ovaries removed. Menopausal status was updated biennially.

#### Oral contraceptive (OC) use

Current and past OC use was asked at 1976 and updated biennially until 1984, at which point no women reported current use. Women were also asked to report the time periods for which they used OCs and reasons for stopping.

#### Parity

Parity, which was defined as pregnancy lasting at least 6 months, was assessed in 1976 and biennially updated through 1984 and updated again in 1996.

### Statistical methods

We used conditional logistic regression to estimate relative risks (RRs) and 95% confidence intervals (CIs), adjusting for potential confounders. We used SAS (version 9.1.3; SAS, Cary, NC) for analyses, and a value of p<0.05 was considered statistically significant. To address the multiple testing issue, in secondary analyses, we calculated adjusted p-values using an optimized false discovery rate approach [30].

Information on exposures and potential confounders was obtained from the biennial questionnaires and was updated through the questionnaire immediately before the diagnosis date of the index case. Potential confounders were family history of glaucoma, body mass index (<22, 22–23.9, 24–25.9, 26–27.9, 28–29.9, ≥30 kg/m^2^), physical activity (quartiles of activity intensity/day), self reported history (yes/no) of diabetes, and cumulatively updated caffeine intake (0–149, 150–299, 300–449, 450–600, ≥600 g/day), age at menopause (20–44, 45–50, 50–54, 54+ years), PMH status (never, past, current user), age at menarche (linear years), parity (linear number of children), and oral contraceptive use (never, ever).

In secondary analyses, we separately analyzed the risk of “high-tension” POAG, defined as those with maximum IOP≥22 mmHg before VF loss (67.5% of all POAG cases) and “normal-tension” POAG, defined as those with maximum IOP<22 mmHg before VF loss.

To evaluate effect modification, we tested the significance of the estimates of the interaction terms from the multivariable conditional logistic regression models, using Wald tests, where the term was constructed as a product of the SNP variables that were coded as 0, 1, 2, and the reproductive factor of interest.

## Results

The characteristics of POAG cases and their matched controls as of the index case’s date of diagnosis were similar (Table 1). Cases had a higher frequency of a family history of glaucoma and self-reported diagnosis of diabetes; however, the cases were somewhat less likely to smoke, drink alcohol or be obese. Among women, the cases were less likely to be current PMH users.

**Table 1:** Characteristics of cases of primary open-angle glaucoma and their matched controls as of diagnosis date.

**Characteristic**	**Cases**	**Controls**
N	374	1085
Age (mean, yr)	64.1	64.0
Family history of glaucoma (%)	35.6	12.2
Diabetes (%)	7.8	5.1
Obesity (Body mass index ≥30 kg/m2, %)	14.0	15.1
Hypertension (%)	37.4	39.0
30+ pack years of smoking (%)	18.2	18.9
Caffeine (mean, mg/day)	313.8	308.0
Alcohol intake (mean, g/day)	6.1	6.2
Number of reported eye exams†	3.1	3.2
Age at menopause (mean, years)	50.1	50.6
Current postmenopausal hormone use (%)	39.8	42.7
Oral contraceptive use (%)	42.5	39.7
Age at menarche (mean, years)	12.6	12.7
Number of children among parous women (mean)	3.3	3.4

†Eye exams have been asked every two years, seven times from 1990- 2002; the number represents the number of eye exams reported as of the period of the diagnosis date of the index case in matched case control sets.

### Summary of main effects of *NOS3* SNPs

The main effects of the *NOS3* polymorphisms in relation to POAG have been previously reported [25]. Briefly, we observed that none of the variants were associated with overall POAG except for rs3918188, where the variant was significantly (p=0.04) inversely associated with overall POAG only among the women (Table 2). In secondary analyses, we found that the T-786C polymorphism was associated with high-tension POAG only among the women (RR=1.80 (95% 1.14, 2.85; p-trend with increasing variant allele=0.02), as was the polymorphism in the tagging SNP rs3918188 (RR=0.48 (95% CI 0.28, 0.82); p-trend with increasing variant allele=0.0008). In relation to normal-tension glaucoma, for the T-786C polymorphism, the “pooled” RR (meta-analyzed RR from NHS and HPFS results) for the CC homozygote was 0.44 (95% CI, 0.22, 0.87) and the p for trend was 0.03.

**Table 2:** Effect modification by female reproductive attributes on the associations of selected *NOS3* polymorphisms and POAG.

**SNP**	**Genotype**	**Overall association between SNP genotypes and POAG**		**Menarche <13 years**	**Menarche ≥13 years**		**Parous 3+ children**	**Parous 1–2 children**		**No oral contraceptive use**	**Oral contraceptive use**
		**RR (95% CI)**	**Number of Cases (MEN-/ MEN+)‡**	**RR (95% CI)§**	**RR (95% CI)§**	**Number of cases (PAR3+/ PAR3-)‡**	**RR (95% CI)§**	**RR (95% CI)§**	**Number of cases (OC-/ OC+)‡**	**RR (95% CI)§**	**RR (95% CI)§**
Promoter −786C/T	TT	1.00 (ref)	67/70	1.00	0.91 (0.59, 1.41)	46/25	1.00	1.20 (0.58, 2.49)	78/59	1.00	1.60 (1.01, 2.55)
	TC	1.11 (0.83, 1.47)	86/80	1.24 (0.82, 1.88)	0.95 (0.62, 1.44)	56/25	0.92(0.54, 1.58)	1.19(0.60, 2.35)	95/71	1.22 (0.84, 1.78)	1.62 (1.04, 2.51)
	CC	1.17 (0.81, 1.70)	34/28	1.33 (0.78, 2.27)	0.99 (0.57, 1.71)	25/7	1.32 (0.67, 2.63)	0.46 (0.15, 1.40)	36/26	1.38 (0.85, 2.27)	1.56 (0.87, 2.81)
					p-int=0.54			p-int=0.13			p-int=0.35
Glu298Asp (rs1799983)	GG	1.00 (ref)	79/85	1.00	1.20 (0.80, 1.81)	49/34	1.00	2.03(1.07, 3.83)	90/74	1.00	1.35 (0.89, 2.04)
	GT	0.98 (0.74, 1.31)	78/65	1.50 (0.99, 2.27)	0.90 (0.59, 1.36)	56/19	1.41 (0.83, 2.41)	1.18 (0.57, 2.44)	89/54	1.16 (0.80, 1.71)	1.22 (0.77, 1.93)
	TT	1.45 (0.98, 2.13)	30/26	2.13 (1.20, 3.77)	1.45 (0.81, 2.59)	19/4	2.01 (0.96, 4.20)	0.51 (0.15, 1.78)	29/27	1.32 (0.78, 2.24)	2.87 (1.50, 5.52)
					p-int=0.05			p-int=0.003¶			p-int=0.60
rs3918188	CC	1.00 (ref)	100/73	1.00	0.54 (0.36, 0.81)	61/19	1.00	0.66 (0.33, 1.32)	98/75	1.00	1.22 (0.80, 1.84)
	CA	0.79 (0.60, 1.03)	73/84	0.57 (0.39, 0.85)	0.56 (0.39, 0.83)	55/30	0.91 (0.54, 1.52)	1.23 (0.67, 2.26)	91/66	0.74 (0.52, 1.06)	0.99 (0.66, 1.48)
	AA	0.69 (0.46, 1.05)	16/25	0.31 (0.16, 0.59)	0.71 (0.40, 1.26)	11/9	0.56 (0.24, 1.33)	1.42 (0.50, 4.01)	24/17	0.59 (0.34, 1.03)	0.90 (0.47, 1.73)
					p-int=0.0007¶			p-int=0.04			p-int=0.59
rs7830	CC	1.00 (ref)	88/84	1.00	0.83 (0.56, 1.23)	54/29	1.00	1.36 (0.70, 2.61)	101/71	1.00	1.22 (0.80, 1.86)
	CA	0.75 (0.57, 0.98)	75/71	0.76 (0.51, 1.14)	0.58 (0.39, 0.86)	52/22	0.73 (0.45, 1.21)	0.75 (0.39, 1.45)	79/67	0.69 (0.48, 0.99)	0.99 (0.66, 1.49)
	AA	1.16 (0.78, 1.73)	27/28	1.04 (0.58, 1.86)	1.09 (0.62, 1.92)	21/7	1.23 (0.59, 2.56)	0.93 (0.32, 2.68)	34/21	1.16 (0.70, 1.93)	1.44 (0.75, 2.75)
					p-int=0.82			p-int=0.29			p-int=0.86

Abbreviations: POAG: primary-open angle glaucoma; OC+: oral contraceptive use, OC-: never used OC; PAR3+: parous with 3 or more children, PAR3-: parous with 1 or 2 children; MEN-: menarche<13, MEN+: menarche≥13. **‡**The number of cases may not add up to 374 because those with missing data were not included in analyses. §Adjusted for family history of glaucoma, hypertension, diabetes, parity, oral contraceptive use, smoking status, body mass index, age at menopause, postmenopausal hormone use, alcohol intake, caffeine intake, physical activity. Statistically significant with multiple testing correction (False Discovery Rate).

### Effect modification with age at menarche

We observed a significant interaction between *NOS3* SNP rs3918188 and age at menarche in POAG. Specifically, the inverse association between the rs3918188 polymorphism and POAG was more evident among those with age at menarche <13 years, after adjustment for age at menopause and other factors (p for interaction=0.0007; significant after multiple testing correction; Table 2). Among those with age at menarche <13 years, compared with the CC homozygotes of the rs3918188 tagging SNP, the AA homozygotes were at significantly reduced risk of POAG (RR=0.31, 95% CI=0.16, 0.59); however, among those with age at menarche ≥13 years, the SNP was not associated with POAG. On the other hand, we did not find a significant interaction between the functional *NOS3* Glu298Asp SNP and age at menarche. Among women with age at menarche <13 years, the adverse association with the Glu298Asp polymorphism was more apparent (RR for TT homozygotes versus GG homozygotes=2.13, 95% CI=1.20, 3.77) than among those with older age at menarche. This interaction was borderline significant (p=0.05), and was no longer significant after multiple testing correction.

### Effect modification with parity

Because 80% of our participants were parous, we did not have the power to examine nulliparity; thus, we examined having 3 or more children versus having 1–2 children for evaluating effect modification. We did observe a significant crossover interaction between the Glu298Asp SNP and parity in POAG. Using women with multiparity who were homozygous for the common Gl298Asp NOS3 variant (GG) as the reference group, both homozygosity for the minor Glu298Asp variant (TT) among those with 3 or more children and homozygosity for the common variant (GG) among women with 1–2 children were adversely related to POAG (p=0.003; significant after multiple testing correction; Table 2). On the other hand, we did not find a significant interaction between the rs3918188 SNP and parity in POAG. Among those with 3 or more children, the protective association with the rs3918188 SNP was more apparent (RR for AA homozygotes versus GG homozygotes=0.56, 95% CI=0.24, 1.33) than among those with 1 or 2 children, but this interaction was borderline significant (p=0.04) and was no longer significant after multiple testing correction.

### Effect modification with oral contraceptives

We did not observe any significant interactions between the four *NOS3* SNPs with oral contraceptive use (Table 2) in relation to POAG.

### Secondary analyses

None of the *NOS3* SNP – female reproductive health attribute showed significant interactions in POAG cases with IOP≥22 mmHg at diagnosis or in POAG cases with IOP≤21 mmHg at diagnosis.

## Discussion

In this nested case-control, we observed that the associations with *NOS3* SNPs in relation to POAG depended on age at menarche and parity, where those associations were most evident among those with greater estrogen exposure (age at menarche <13 years and parity of 3 or more children versus 1–2 children). Interactions were not observed with oral contraceptives in relation to POAG overall. Because this is the first study to examine the interaction between *NOS3* SNPS and female reproductive factors, these results need to be confirmed in other studies.

These findings should be interpreted in the context of previous cohort studies describing the relation between reproductive factors and POAG [24,25,31]. For age at menarche, in the NHS, we previously found that menarche >13 years was associated with increased risk of POAG with IOP≤21 mmHg at diagnosis [31], which was consistent with the finding from the Blue Mountain Eye Study that reported that later age at menarche was associated with an increased risk of OAG [32]. These data suggest that increased lifetime estrogen exposure during the reproductive years, as reflected in earlier menarche, is associated with POAG. Among the *NOS3* SNPs, the functional consequences of the rs3918188 tagging SNP are unknown; however, we previously found that the minor allele (A) was associated with a reduced risk of POAG in women (p-trend=0.04) but not men (p-trend=0.58). We observed a significant interaction between the minor allelic variant of the rs3918188 tagging SNP and age at menarche in POAG: women with homozygosity for the minor allele and age at menarche <13 years had a 69% reduction in POAG risk compared to those with homozygosity for the wild-type allele and older age at menarche. Biologically, this is plausible as increased age at menarche may be related to reduced NOS3 activity [33], which may also reduce endothelial cell relaxation in the trabecular meshwork cells and in the vascular endothelium that supply retinal ganglion cells. In addition, we previously observed significant interactions between this same SNP and current PMH use in POAG with IOP>21 mmHg at diagnosis: postmenopausal women with homozygosity for the minor allele and current users of PMH had a 67% reduced risk of high tension POAG.

We also observed that the association between the functional Glu298Asp SNP and POAG was significantly modified by parity. For parity, in the NHS, we reported a null association with POAG [31], although the Blue Mountain Eye Study observed that increasing parity was associated with an increased risk of OAG [33]. For the functional Glu298Asp SNP, which is known to reduce intercellular nitric oxide levels [34,35], we previously did not find an association with POAG in women or in men, nor any interactions with age at menopause or PMH use. Here, we observed a “cross-over” interaction where the wild type genotype was associated with increased POAG risk for women with parity <3, while the dysfunctional genotype was associated with increased risk for women with parity 3+. Multiparity is known to produce endothelial dysfunction through reduced nitric oxide bioavailability [36], thus, it is plausible that multiparity-induced endothelial dysfunction may be exacerbated in homozygotes of the dysfunctional NOS3 variant. The adverse association with the common SNP among those with lower parity is relative to women of the same genotype who had more children. Perhaps in the latter instance lower circulating estrogen milieu in women with low parity may be more important than multiparity-induced endothelial dysfunction in dictating POAG risk. We have previously reported a “cross-over” type interaction for the functional T-786C SNP and hypertension and POAG [37]. It remains to be determined whether crossover interactions have real pathophysiological significance.

The associations between the *NOS3* SNPs and reproductive factors that reflect exposure to estrogen, which potently modulates *NOS3* activity, provides further support for the important role of endothelial cell dysfunction in POAG etiology. Such dysfunction in the trabecular meshwork and ocular vascular endothelium may lead to increased IOP and retinal ganglion cell vulnerability, resulting in glaucomatous visual loss. Recent emerging data from animal models, studies of POAG genetics and clinical trials of agents related to vascular tone also support this hypothesis. In a mouse model, there is evidence that knocking out soluble guanylate cyclase, the downstream mediator of nitric oxide, produces modest increases in IOP and considerable loss of retinal ganglion cells and nerve fiber layer dropout [38]. In an Icelandic genome-wide association study of POAG, gene variants in the intergenic region between caveolin 1 and caveolin 2, which code for caveolins that reside near NOS3 in endothelial cell membranes and serve to mediate endothelial tone were associated with POAG [39]. A randomized clinical trial found that topical brimonidine, an alpha 2 agonist with vasomotor activity at the level of the retinal vascular endothelial cells, was superior to timolol, in preserving vision in the normal tension variant of POAG [40-43]. Other studies of statins, which also improve endothelial cell function, have found that they may favorably alter the course of POAG [44,45]. Thus, our data, in combination with emerging data from laboratory as well as human studies, coherently implicate endothelial cell dysfunction in POAG pathogenesis.

There are limitations to this work that should be considered. First, our glaucoma definition was based on self-report with confirmation with medical records and visual fields. This definition has very high specificity, as we required documentation of reproducible defect on at least 2 reliable visual field tests and we have demonstrated strong associations with established risk factors, such as African-American heritage and family history [28]. Given the insidiousness of glaucoma, some controls might have had undiagnosed glaucoma. However, it is unlikely to have had a major influence on our results, as the prevalence of glaucoma in adults over age 40 is 1.3% in Caucasians [46]. Furthermore, our controls had an eye exam as of the matched cases’ diagnosis dates. In fact, the average number of eye exams reported as of their selection as controls were three exams, implying that manifest glaucoma, if present, would likely have been detected. Any misclassifications of the disease would have biased the results toward the null. Third, our participants were generally healthy Caucasians and thus we are unable to make inferences to less healthy populations or minorities. Finally, it is possible that our results may be due to chance, despite the fact we showed statistical significance after accounting for multiple comparisons. Therefore, these findings should be interpreted with caution and confirmed in future studies, particularly with different racial/ethnic groups.

In conclusion, the associations between *NOS3* SNPs and POAG depended on parity and age at menarche. Previously we showed the associations between *NOS3* SNPs and POAG depended on PMH use. These data indicate that *NOS3* gene variants interact with sex hormone levels in ways to influence the risk of POAG. In the future we hope to validate these interactions in animal model systems.
